# Intersurgeon Variability in Local Treatment Planning for Patients with Initially Unresectable Colorectal Cancer Liver Metastases: Analysis of the Liver Expert Panel of the Dutch Colorectal Cancer Group

**DOI:** 10.1245/s10434-023-13510-7

**Published:** 2023-04-28

**Authors:** Marinde J. G. Bond, Babette I. Kuiper, Karen Bolhuis, Aysun Komurcu, Martinus J. van Amerongen, Thiery Chapelle, Cornelis H. C. Dejong, Marc R. W. Engelbrecht, Michael F. Gerhards, Dirk J. Grünhagen, Thomas van Gulik, John J. Hermans, Koert P. de Jong, Joost M. Klaase, Niels F. M. Kok, Wouter K. G. Leclercq, Mike S. L. Liem, Krijn P. van Lienden, I. Quintus Molenaar, Ulf P. Neumann, Gijs A. Patijn, Arjen M. Rijken, Theo M. Ruers, Cornelis Verhoef, Johannes H. W. de Wilt, Geert Kazemier, Anne M. May, Cornelis J. A. Punt, Rutger-Jan Swijnenburg

**Affiliations:** 1 Department of Epidemiology, Julius Centre for Health Sciences and Primary Care, University Medical Centre Utrecht, Utrecht University, Utrecht, The Netherlands; 2 Department of Surgery, Amsterdam UMC, University of Amsterdam and Vrije Universiteit Amsterdam, Amsterdam, The Netherlands; 3 Department of Medical Oncology, Amsterdam UMC, Cancer Centre Amsterdam, Amsterdam, The Netherlands; 4 Department of Gastrointestinal Oncology, Netherlands Cancer Institute, Amsterdam, The Netherlands; 5 The Netherlands Comprehensive Cancer Centre, Utrecht, The Netherlands; 6 Department of Radiology, Sint Maartenskliniek, Nijmegen, The Netherlands; 7 Department of Hepatobiliary, Transplantation, and Endocrine Surgery, Antwerp University Hospital, Antwerp, Belgium; 8 Department of Surgery, Maastricht University Medical Centre, Maastricht, The Netherlands; 9 Universitätsklinikum Aachen, Aachen, Germany; 10 Department of Radiology and Nuclear Medicine, Amsterdam UMC, Amsterdam, The Netherlands; 11 Department of Surgery, OLVG Hospital, Amsterdam, The Netherlands; 12 Department of Surgery, Erasmus MC Cancer Institute, Rotterdam, The Netherlands; 13 Department of Radiology, Radboud University Medical Centre, Nijmegen, The Netherlands; 14 Department of Hepatobiliary Surgery and Liver Transplantation, University Medical Centre Groningen, Groningen, The Netherlands; 15 Department of Surgery, Netherlands Cancer Institute, Amsterdam, The Netherlands; 16 Department of Surgery, Máxima Medical Centre, Veldhoven, The Netherlands; 17 Department of Surgery, Medical Spectrum Twente, Enschede, The Netherlands; 18 Department of Radiology, Sint Antonius Hospital, Nieuwegein, The Netherlands; 19 Department of Surgery, University Medical Centre Utrecht, Utrecht, The Netherlands; 20 Department of Surgery, Isala Hospital, Zwolle, The Netherlands; 21 Department of Surgery, Amphia Hospital, Breda, The Netherlands; 22 Department of Surgery, Radboud University Medical Centre, Nijmegen, The Netherlands; 23 Cancer Center Amsterdam, Amsterdam, The Netherlands

## Abstract

**Background:**

Consensus on resectability criteria for colorectal cancer liver metastases (CRLM) is lacking, resulting in differences in therapeutic strategies. This study evaluated variability of resectability assessments and local treatment plans for patients with initially unresectable CRLM by the liver expert panel from the randomised phase III CAIRO5 study.

**Methods:**

The liver panel, comprising surgeons and radiologists, evaluated resectability by predefined criteria at baseline and 2-monthly thereafter. If surgeons judged CRLM as resectable, detailed local treatment plans were provided. The panel chair determined the conclusion of resectability status and local treatment advice, and forwarded it to local surgeons.

**Results:**

A total of 1149 panel evaluations of 496 patients were included. Intersurgeon disagreement was observed in 50% of evaluations and was lower at baseline than follow-up (36% vs. 60%, *p* < 0.001). Among surgeons in general, votes for resectable CRLM at baseline and follow-up ranged between 0–12% and 27–62%, and for permanently unresectable CRLM between 3–40% and 6–47%, respectively. Surgeons proposed different local treatment plans in 77% of patients. The most pronounced intersurgeon differences concerned the advice to proceed with hemihepatectomy versus parenchymal-preserving approaches. Eighty-four percent of patients judged by the panel as having resectable CRLM indeed received local treatment. Local surgeons followed the technical plan proposed by the panel in 40% of patients.

**Conclusion:**

Considerable variability exists among expert liver surgeons in assessing resectability and local treatment planning of initially unresectable CRLM. This stresses the value of panel-based

decisions, and the need for consensus guidelines on resectability criteria and technical approach to prevent unwarranted variability in clinical practice.

**Supplementary Information:**

The online version contains supplementary material available at 10.1245/s10434-023-13510-7.

Local treatment (e.g., surgery, ablation) is the only potentially curative treatment for patients with colorectal cancer liver metastases (CRLM). Survival rates of 30–50% have been reported for patients with initially unresectable CRLM who received local treatment.^1^ Results from clinical trials show that 11–57% of patients with initially unresectable CRLM convert to resectable CRLM after downsizing by systemic therapy.^2^ A major complicating factor in the interpretation of these results is the lack of consensus on criteria for (un)resectability of CRLM. Consequently, there is a subjective and therefore variable component in the decision-making process, which is also highly dependent on the multidisciplinary treatment options in different treatment centres. This is illustrated by a previous study in which experienced liver surgeons were asked to choose a treatment strategy in ten different CRLM patients, where disagreement on therapeutic strategies was observed in most cases.^3^ Another key issue is that not all patients who are eligible for local treatment of CRLM are referred to dedicated liver centres to be offered this option.^4–7^ Previous studies have shown that local treatment rates differ according to the treatment setting, to the potential detriment of patients who are not treated in liver-dedicated centres.^8,9^ Lack of resection criteria and low referral rates could be resolved by using easily accessible online expert panels, which, according to two retrospective studies, results in higher rates of patients eligible for local treatment.^10,11^ However, disagreement among experienced liver surgeons was also present in liver expert panels when assessing resectability in clinical trials:^12,13^ disagreement was observed in 52% of the resectability assessments as reported by a previous evaluation of the Dutch Colorectal Cancer Group (DCCG) liver expert panel.^13^ The online DCCG liver expert panel, consisting of experienced liver surgeons and abdominal radiologists, prospectively assessed (un)resectability in the CAIRO5 study, in which the currently most effective induction regimens in patients with initially unresectable CRLM are compared.^14^ The current study is an extension of the previous evaluation and was conducted for three reasons. Firstly, the increased sample size and experience of the DCCG liver expert panel allows for a more robust analysis. Secondly, while the variability in resectability assessments among surgeons at the level of individual patients has been investigated, the general variability remains unknown between individual surgeons in assessing resectability. Thirdly, the DCCG liver expert panel also provides technical local treatment plans for patients evaluated as having resectable CRLM, and the preferences of surgeons for certain strategies have not been examined before. Strategies to achieve clearance of all CRLM include one-stage minor or major liver resections, combinations of local resections with tumour ablation or stereotactic body radiation therapy (SBRT), two-stage resections with or without preoperative portal vein embolisation, and/or associating liver partition and portal vein ligation for staged hepatectomy (ALPPS).

The aim of the current study was to assess the variability among liver surgeons: those participating in the DCCG liver expert panel, in resectability assessments and in local treatment planning in patients with initially unresectable CRLM receiving induction systemic therapy.

## Methods

### Patient Selection

Patients were selected from the CAIRO5 study (NCT02162563), a randomised phase III trial of the DCCG, comparing the currently most effective systemic induction regimens in patients with initially unresectable colorectal cancer liver-only metastases.^14–16^ Patients randomised between the start of the study in November 2014 and April 2021 were selected for this subset analysis. The CAIRO5 study was conducted in accordance with the standards of Good Clinical Practice and the Declaration of Helsinki. The CAIRO5 study was approved by the local ethics committees and all patients provided written informed consent before the start of the study.

### Resectability Assessment

The DCCG online liver expert panel, currently consisting of 15 experienced liver surgeons and 3 abdominal radiologists, evaluated unresectability at baseline and resectability every 2 months during follow-up. Given the lack of consensus on (un)resectability criteria, baseline resectability criteria were established by liver surgeons from the expert panel, resulting in clear entry criteria allowing for a homogeneous study population. CRLM was considered unresectable at baseline if an R0 resection could not be achieved with surgical resection only in one stage. Resectability (or amenability to local treatment) during follow-up was based on more liberal resection criteria, since all established local treatments were allowed (i.e., ablation, two-stage surgery, portal vein embolisation) to achieve clearance of all CRLM while preserving a functional liver remnant. The design of the panel has previously been described in detail.^13^ In short, after evaluation by one radiologist, each CT scan with panel radiology report [including patient’s age, number of treatment cycles, location and resection (yes/no) of primary tumor] was evaluated by three randomly selected panel surgeons, who voted individually on the following categories: resectable, potentially resectable after further induction systemic treatment, or permanently unresectable. Permanently unresectable was selected when there was expected failure of achieving a complete R0 resection or ablation of all CRLM at any moment during systemic therapy. If no consensus (i.e., same category selected by all three surgeons) was obtained, two additional surgeons were consulted by the panel chair (consecutively T.v.G., J.K., R.S.) and the majority vote was accepted by the panel chair as the final vote. If the vote was 2 vs 2 vs 1, the panel chair determined the vote. During follow-up, patients with permanently unresectable CRLM as the final vote were not re-evaluated by the panel. If panel surgeons voted for resectable CRLM, they were asked to provide a detailed technical plan for their local treatment approach. The following items were included in the technical plan: modality [wedge resection/segmental resection/ablation/(extended) hemihepatectomy] specified per segment, one- or two-stage approach, portal vein embolisation (no/yes + left/right). The panel chair decided on one final technical plan, based on the plans of the other panel surgeons. The panel conclusion was forwarded to the referring hospital, along with the proposed local treatment advice if the CRLM was resectable.

### Outcomes

The degree of agreement among surgeons has previously been described in detail.^13^ In short, minor disagreement was defined as a panel evaluation in which at least one panel surgeon assessed the CRLM as potentially resectable and at least one other surgeon in the same panel voted for resectable CRLM, or a combination of potentially resectable and permanently unresectable CRLM. Major disagreement was defined as a panel evaluation in which at least one panel surgeon assessed the CRLM as resectable and at least one other surgeon voted for permanently unresectable. Intersurgeon variability per individual patient applies to the differences among surgeons in the assessment of the same patient. Intersurgeon variability in general refers to differences among surgeons considering all patients they assessed. In the latter analyses, surgeons with fewer than 10 observations were excluded and the evaluations of four former panel surgeons were included as well. Local treatment plans were considered similar if all surgeons proposed the same type of treatment as presented in Supplementary Table S1, and different if at least one surgeon proposed a different plan. No distinction was made between which segment was treated with which modality because of the clinical relevance (e.g., if one surgeon proposed a wedge resection of a lesion in segment II and ablation of a lesion in segment IV, and the other surgeon proposed the reverse, the plan was considered similar). Complete local treatment was defined as complete R0/R1 resection or ablation of all CRLM. SBRT for a remaining lesion was allowed to qualify for complete local treatment.

### Statistical Analysis

Continuous variables were displayed as median with interquartile range (IQR) and categorical variables as counts and percentages. Differences between groups were analysed using Pearson's chi-square test and Fisher exact test, as appropriate. A *p* value ≤ 0.05 was considered statistically significant. All analyses were performed in R (version 4.0.3).

## Results

In total, 1149 panel evaluations were analysed [494 (43%) baseline evaluations and 655 (57%) follow-up evaluations] of 494 patients. Patient characteristics are presented in Table 1. The median time to panel conclusion at baseline improved from 6 days (IQR 4–10) in the first year to 3 days (IQR 2–6) in the last year and at follow-up from 9.5 days (IQR 7–12) to 4 days (IQR 2–7). The evaluations of 17 surgeons from 13 different medical centres were used to assess the general intersurgeon variability. The median time of experience of these surgeons was 22 years (IQR 18–24). The hospitals where the liver surgeons worked performed a median number of 79 liver resections (IQR 63–98) annually, as calculated over 2019–2021 based on data from the Dutch Hepato Biliary Audit.

**Table 1:** Patient characteristics

	*N* = 494
Age	62 (54–69)
Sex	
Female	187 (37.9%)
Male	307 (62.1%)
WHO performance status	
0	317 (64.2%)
1	174 (35.2%)
2	2 (0.4%)
Unknown	1 (0.2%)
Primary tumour location	
Left	361 (73.1%)
Right	133 (26.9%)
Time to metastasis	
Metachronous	54 (10.9%)
Synchronous	440 (89.1%)
*RAS* mutation	
No	244 (49.4%)
Yes	250 (50.6%)
*BRAF*^*V600E*^ mutation	
No	464 (93.9%)
Yes	30 (6.1%)
Number of liver metastases	12 (7–22)
Size of largest liver metastasis (millimetres)	42 (27–65)
Diaphragm involved	
Yes	187 (37.9%)
No	270 (54.7%)
Unknown	37 (7.5%)
Vena cava involved	
Yes	165 (33.4%)
No	312 (63.2%)
Unknown	17 (3.4%)
Hepatic vein involved	
Yes	345 (69.8%)
No	136 (27.5%)
Unknown	13 (2.6%)
Hepatic artery involved	
Yes	71 (14.4%)
No	270 (54.7%)
Unknown	153 (31.0%)
Portal vein involved	
Yes	241 (48.8%)
No	240 (48.6%)
Unknown	13 (2.6%)

### Intersurgeon Variability per Individual Patient in Resectability Assessments

Overall, consensus among panel surgeons was observed in 578 (50%) evaluations, minor disagreement in 456 (40%) and major disagreement in 115 (10%). In the 324 patients considered to have resectable CRLM by the panel, consensus was observed in 141 (44%) evaluations, minor disagreement in 131 (40%) and major disagreement in 52 (16%).

The degree of agreement among resectability assessments per evaluation point is presented in Supplementary Fig. S1. Any intersurgeon disagreement (minor or major) was lower at baseline compared with follow-up panel evaluations [179 (36%) vs. 392 (60%), *p* < 0.001]. Major intersurgeon disagreement was lower at baseline compared with follow-up panel evaluations [4 (1%) vs. 111 (17%), *p* < 0.001]. No difference was observed in overall disagreement over time, neither when major and minor disagreement were grouped together as any disagreement (*p* = 0.091), nor when split into minor and major disagreement (*p* = 0.370) (Supplementary Fig. S2).

### Intersurgeon Variability per Individual Patient in Technical Local Treatment Planning

**Fig. 1:**
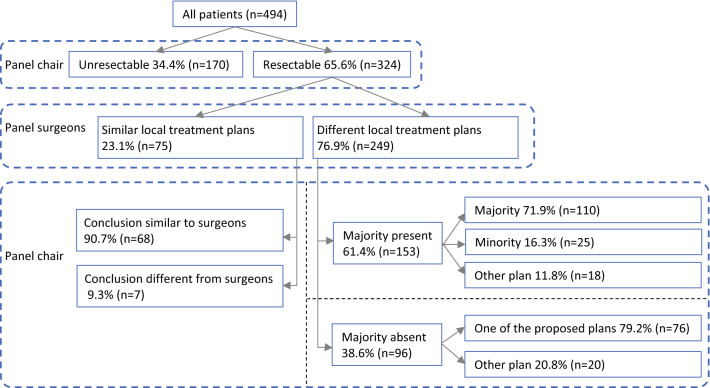
Resectability and local treatment plans. The definition of the majority depended on the number of surgeons who voted for resectable CRLM. Two surgeons: majority absent. Three surgeons: at least two similar plans. Four surgeons: at least three similar plans

The panel considered 324 (66%) patients to have resectable CRLM (Fig. 1). In 75 (23%) of these patients, the panel surgeons proposed a similar local treatment plan, which was adopted by the chair in 91% (68/75). In 249 (77%) of these patients, surgeons proposed different local treatment plans. A majority of surgeons proposing a similar plan was present in 153 (61%) of these patients. The chair adopted the majority’s plan in 110/153 (72%) patients, followed one of the other panel surgeons’ plans (i.e., minority) in 25/153 (16%) patients and proposed a completely different plan in 18/153 (12%) patients. In the absence of a majority [96/249 (39%) patients], the chair followed one of the proposed plans in 76 patients (79%) and created a new plan in 20 patients (21%).

The differences between the surgeons’ plans are detailed in Table 2. The major differences in local treatment were: one or more surgeons proposing a parenchymal-preserving approach with local resection and/or ablation versus one or more surgeons proposing a hemihepatectomy (± local resection/ablation) in a one-stage approach [75/249 patients (30%)] or in a two-stage approach [31/249 patients (12%)], and a one-stage versus two-stage hemihepatectomy (± local resection/ablation) [68/249 patients (27%)].

**Table 2:** Differences between the proposed plans by the panel surgeons

	*N* = 249
Different strategies of local resection/ablation	22 (8.8%)
Different strategies of local resection/ablation + HH	10 (4.0%)
Different strategies of two-stage local resection/ablation + HH	18 (7.2%)
Local resection/ablation versus one-stage incl. HH	75 (30.1%)
Local resection/ablation versus two-stage incl. HH	31 (12.4%)
One-stage versus two-stage	68 (27.3%)
Mix of local resection/ablation and one- and two-stage incl. HH	25 (10.0%)

‘Different strategies of local resection/ablation’ includes all possible combinations of wedge resections, segmental resections and/or ablation (e.g., two surgeons proposed a wedge resection and one surgeon proposed a segmental resection). The second and third categories are similar to the first category, but these also include a (two-stage) hemihepatecomy. Another example: ‘local resection/ablation versus one-stage incl. HH’ could be based on one surgeon proposing a combination of segmental resections and ablation and three surgeons proposing ablation combined with a hemihepatectomy. *HH* = hemihepatectomy; *Local resection* = wedge resection and/or segmental resection

### General Intersurgeon Variability in Resectability Assessments

**Fig. 2:**
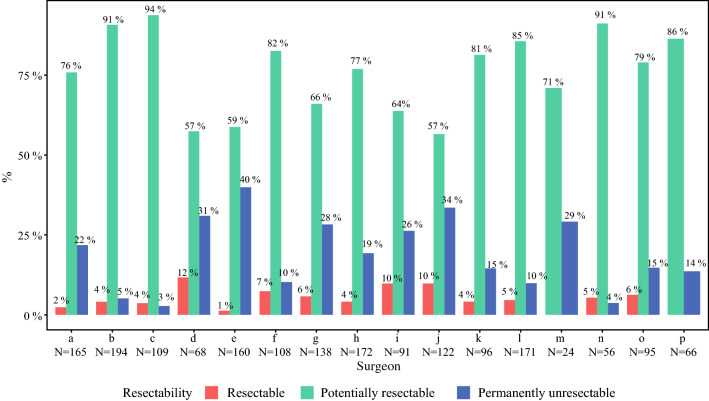
Assessment of resectability by individual surgeons at baseline. In total there were 1836 resectability assessments by panel surgeons at baseline, of which 1835 are represented in this figure (one surgeon with one evaluation was excluded)

At baseline, there were 1836 resectability assessments by panel surgeons in 494 patients. In 1400 (76%) resectability assessments surgeons voted for potentially resectable CRLM, in 91 (5%) for resectable CRLM and in 345 (19%) for permanently unresectable CRLM at baseline. Votes for permanently unresectable CRLM at baseline decreased from 33 to 13% in the first to last 20% of resectability assessments, respectively. The resectability assessments per surgeon at baseline are depicted in Fig. 2. Votes per surgeon for resectable CRLM ranged between 0 and 12% (surgeons M and D) and for permanently unresectable CRLM between 3 and 40% (surgeons C and E).

**Fig. 3:**
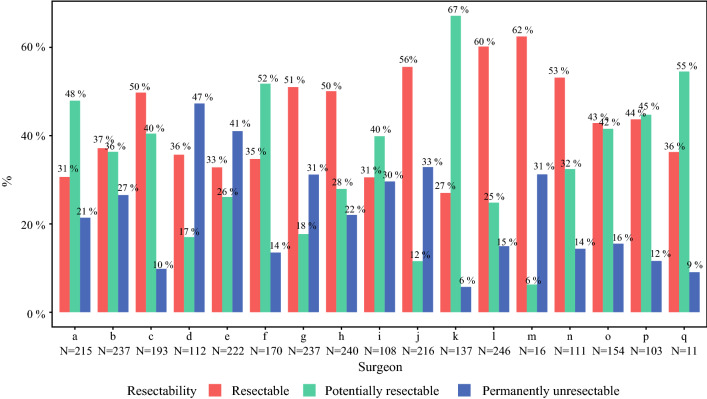
Assessment of resectability by individual surgeons at follow-up**.** In total there were 2728 resectability assessments by panel surgeons at follow-up

During follow-up, there were 2728 resectability assessments by panel surgeons (in 481 patients at first, 144 at second, 27 at third and 3 at fourth follow-up). In 917 (34%) resectability assessments surgeons voted for potentially resectable CRLM, in 1185 (43%) for resectable CRLM and in 628 (23%) for permanently unresectable CRLM during follow-up. Votes for permanently unresectable CRLM during follow-up decreased from 30 to 19% in the first to last 20% of resectability assessments, respectively. The resectability assessments per surgeon during follow-up are depicted in Fig. 3. Votes for resectable CRLM ranged between 27 and 62% (surgeons K and M) and for permanently unresectable CRLM between 6 and 47% (surgeons K and D).

### General Intersurgeon Variability in Technical Local Treatment Planning

**Fig. 4:**
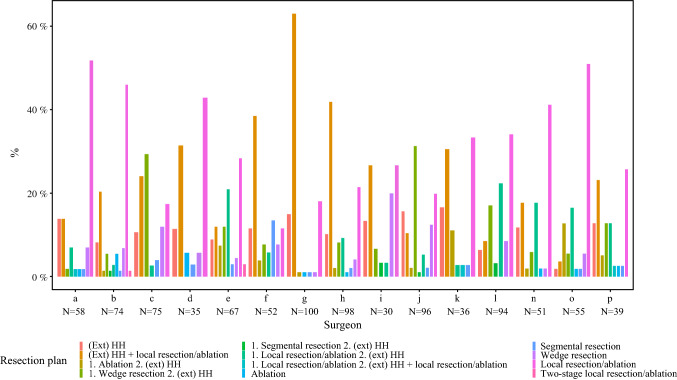
Local treatment plans proposed by individual surgeons in patients with resectable CRLM. In total there were 972 proposed plans, of which 12 plans by two surgeons were excluded because they evaluated < 10 cases. *Ext* = extended; *HH* = hemihepatectomy; *local resection* = wedge resection and/or segmental resection

Figure 4 shows the proposed technical plans of all surgeons for patients amenable to local treatment. In total there were 972 assessments with a vote for resectable CRLM which included a technical treatment plan. In general, there was a high variability among surgeons. The largest difference consisted of surgeons who proposed a hemihepatectomy ± local resection/ablation [4–63% (surgeons O and G)] versus surgeons who proposed a parenchymal-preserving approach with local resection and/or ablation [12–52% (surgeons F and A)].

### Adherence by the Local Treatment Centre to the Panel Advice

Local treatment of CRLM was performed in 271 of 324 (84%) patients evaluated by the panel as having resectable CRLM and this was complete (R0/R1 resection and/or ablation) in 235 (87%) patients. CRLM of 16 patients (5%) was considered perioperatively unresectable and 37 (11%) received no local treatment. Reasons for decisions to withhold local treatment are presented in Table 3. Six out of a hundred and seventy patients (4% of all unresectable CRLM) received local treatment against panel advice and 10/170 (6%) patients before the panel assessment of which 12/16 (75%) local treatments were complete. This resulted in a rate of 58% (287/494) for attempted local treatment and 50% (247/494) for complete local treatment.

**Table 3:** Reasons why no local treatment was performed in patients with CRLM that was resectable according to the panel

	*N* = 37
Progressive disease	
Liver	5 (13.5%)
Extrahepatic	6 (16.2%)
Both	1 (2.7%)
Decision of local surgeon/oncologist/MDT	12 (32.4%)
Condition of patient	5 (13.5%)
Decision of patient	2 (5.4%)
In retrospective already lung metastases	2 (5.4%)
SBRT only	1 (2.7%)
Unknown	3 (8.1%)

*MDT* Multidisciplinary team; *SBRT* Stereotactic body radiation therapy

Local treatment was performed after a median of 6 (range 3–15) cycles of systemic therapy and after a median time of 48 days (range 0–243) after the panel conclusion was reached.

The type of local treatment in patients who received complete local treatment is shown in Supplementary Table S1. The most commonly performed strategy was a combination of local resection and ablation (41%), followed by a one-stage (extended) hemihepatectomy combined with local resection and/or ablation (13%). A two-stage approach was performed in 49 patients (21%), of which 12 were formal ALPPS procedures. Four patients with complete local treatment also received radiotherapy for a lesion which could not be treated with resection or ablation.

In 94/235 (40%) patients, the proposed treatment plan by the panel chair was followed by the local surgeon in the referring hospital. The differences between performed and advised local treatment are shown in Table 4. In 35% of patients, the local surgeon chose a more parenchymal-preserving approach by performing a combination of local resection and/or ablation instead of the advised hemihepatectomy (± local resection/ablation) in a one- or two-stage approach [31/141 (22%) and 18/141 (13%) patients, respectively]. The opposite was observed in 12%, where the panel chair advised a parenchymal-preserving approach while the local surgeon performed a hemihepatectomy (± local resection/ablation) one- or two-staged [9/141 (6%) and 8/141 (6%) patients, respectively].

**Table 4:** Differences between local treatment performed by the local surgeon versus the proposed plan by the panel chair

		*N* = 141
Different strategies of local resection/ablation		26 (18.4%)
Different strategies of local resection/ablation + HH		9 (6.4%)
Different strategies of two-stage local resection/ablation + HH		18 (12.8%)
*Performed by local surgeon*	*Proposed by panel chair*	
HH ± local resection/ablation	Local resection/ablation	9 (6.4%)
Local resection/ablation	HH ± local resection/ablation	31 (22.0%)
Two-stage local resection/ablation + HH	Local resection/ablation	8 (5.7%)
Local resection/ablation	Two-stage local resection/ablation + HH	18 (12.8%)
Two-stage local resection/ablation + HH	HH ± local resection/ablation	13 (9.2%)
HH ± local resection/ablation	Two-stage local resection/ablation + HH	4 (2.8%)
Three-stage	Two-stage	2 (1.4%)
Two-stage local resection/ablation	Local resection/ablation	1 (0.7%)
Two-stage local resection/ablation	HH ± local resection/ablation	1 (0.7%)
Two-stage local resection/ablation	Two-stage local resection/ablation + HH	1 (0.7%)

‘Different strategies of local resection/ablation’ includes all possible combinations of wedge resections, segmental resections, and ablation (e.g., the panel proposed a wedge resection and the local surgeon performed a segmental resection). The second and third categories are similar to the first category, but these also include a (two-stage) hemihepatecomy. *HH* = hemihepatectomy; *Local resection* = wedge resection and/or segmental resection

## Discussion

This study showed considerable intersurgeon variability in resectability assessments and in technical local treatment planning for patients with initially unresectable CRLM receiving induction systemic therapy. A plausible explanation for the higher amount of disagreement at follow-up compared with baseline may be that the (un)resectability criteria at baseline were strictly defined but more liberal at follow-up. The increasing disagreement rate from the first to the last follow-up may be explained by the fact that patients with CRLM that are technically challenging to treat remain in the follow-up process. Adherence to resectability assessments was high, with 84% of patients who were considered to have resectable CRLM by the panel actually receiving local treatment. In contrast, the variability in technical treatment plans was high (77%) and adherence to the proposed technical local treatment plans by referring surgeons was low (40%).

The general intersurgeon variability in prospective resectability assessments of multiple surgeons over several years has not been investigated before. The observed differences are to be expected considering, first, the large variation in resection rates for CRLM between hospital types and regions,^8,9,17^ and second, the lack of consensus on resectability criteria, with expanding indications and local treatment modalities, and intensification of systemic therapy during recent years, which is also reflected by the decreasing proportion of votes for permanently unresectable CRLM. Limitations in the surgeon’s technical capacities or different views on the effectiveness of local ablation in certain subgroups of patients are possible explanations for different views on resectability. Apart from the variability in resectability assessments, a large amount of intersurgeon variability in technical plans for local treatment was observed. The most relevant difference is that some surgeons appeared to have a clear preference for a hemihepatectomy (± local resection/ablation), while others preferred a parenchymal-preserving approach with a combination of local resection and/or ablation. Part of the variability in technical plans may be explained by the preference of some surgeons for local or major liver resection over ablation due to the alleged higher risk of local recurrence and potentially poorer oncological outcomes. However, unbiased high-quality evidence is lacking, and the non-inferiority of ablation to local resection is being investigated in ongoing randomised controlled trials.^18,19^

Numerous strategies can be followed to achieve clearance of CRLM, and it is currently not known which strategy is the most beneficial for patients. A previous systematic review comprising retrospective studies demonstrated better perioperative outcomes without compromising oncological outcomes with the use of a parenchymal-preserving approach compared with a hemihepatectomy.^20^ However, these results may not be generalisable due to the inability of ruling out selection bias, and the varying definitions of a parenchymal-preserving approach complicates the interpretation of the results. Upon completion of the CAIRO5 study,^14^ it will be possible to evaluate a possible correlation between perioperative and oncological outcomes and the various strategies as proposed by the panel.

The large amount of intersurgeon variability reflects the complexity of defining local treatment strategies for patients with CRLM. Variability in clinical practice may reflect differences between patient characteristics or well-informed preferences of patients.^21^ However, the strength of this study is that surgeons were randomly assigned to evaluate patients from a homogeneous trial population, therefore it is unlikely that the observed variability is caused by differences between patients. Hence, the observed variability should be considered unwarranted and efforts should be made to reduce this in order to ensure that all patients have the same probability of receiving curative-intent local treatment regardless of which hospital they are treated in. To reduce the unwarranted variability, consensus guidelines on resection criteria and technical approach are warranted, and the use of an expert panel should be advocated, which is supported by previous studies.^8–13,22^ The short time between uploading imaging by the referring centres and reaching a panel conclusion shows that the use of an online expert panel does not cause a significant delay in treatment initiation, and is feasible. We suggest simplifying a future liver expert panel by focusing on the resectability assessment (resectable, potentially resectable, permanently unresectable) and either include all proposed plans instead of one final plan formed by the panel chair, or omit the technical treatment plans to reduce the workload without compromising the objective. Proposing technical treatment plans seems of limited value since a high variability among the panel surgeons and a low adherence to the plans by the referring surgeons was observed. The low adherence may partly be explained by the lack of information on volume or function of the future liver remnant for the panel, which may be available to the local surgeon and plays an important role in the choice of the technical approach. Additionally, the treatment plan may be influenced by the preference and condition of a patient or pre- or perioperative new findings.

Future research should be directed towards evaluating whether the level of disagreement and/or the complexity of treatment strategies correlate with clinical outcomes. In addition, further research is needed to determine whether panel evaluations may be supported by biological resection criteria, such as the consensus molecular subtypes and circulating tumour DNA, to select patients with CRLM who will derive the most benefit from local treatment.

## Supplementary Information

Below is the link to the electronic supplementary material.

Supplementary file1 (PDF 256 KB)

## Data Availability

The data that support the findings of this study are available from the corresponding author upon reasonable request.
