# Supplementary material for: Intersurgeon Variability in Local Treatment Planning for Patients with Initially Unresectable Colorectal Cancer Liver Metastases: Analysis of the Liver Expert Panel of the Dutch Colorectal Cancer Group
Source: Ann Surg Oncol. 2023 Apr 28;30(9):5376–85. doi: 10.1245/s10434-023-13510-7 (PMC10409679; doi:10.1245/s10434-023-13510-7)
Supplement: Supplementary file 1 — Supplementary file1 (PDF 256 KB) [file 10434_2023_13510_MOESM1_ESM.pdf]

Figure S1: The degree of agreement among surgeons per individual patient in resectability evaluations per evaluation moment. Minor disagreement = combination of potentially resectable and resectable, or potentially resectable and permanently unresectable. Major disagreement = ≥1 resectable and ≥1 permanently unresectable.

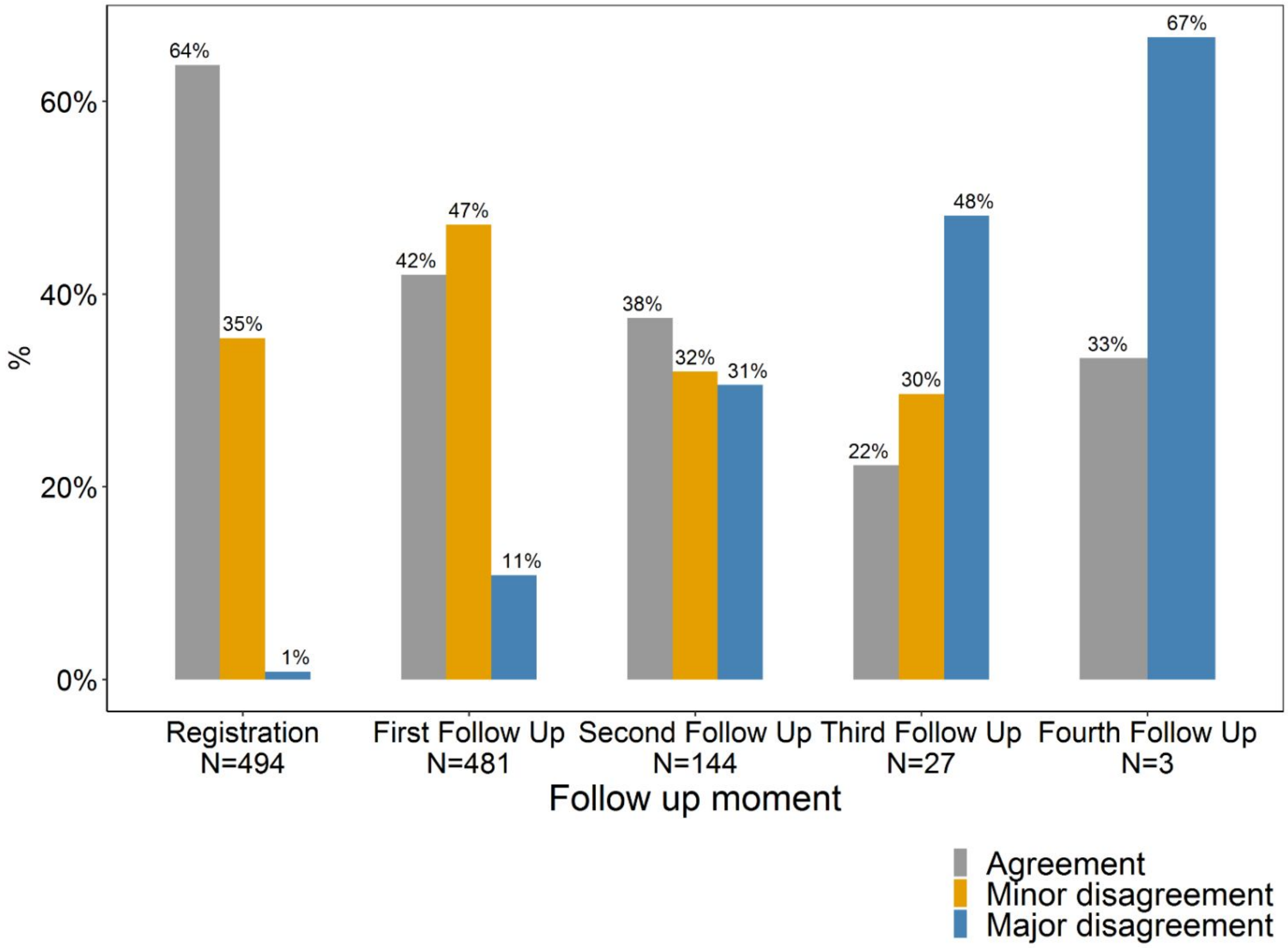

Figure S2: The degree of agreement among surgeons per individual patient in resectability evaluations over time. Patients were divided into six chronological groups to show the variability over time. Minor disagreement = combination of potentially resectable and resectable, or potentially resectable and permanently unresectable. Major disagreement = ≥1 resectable and ≥1 permanently unresectable.

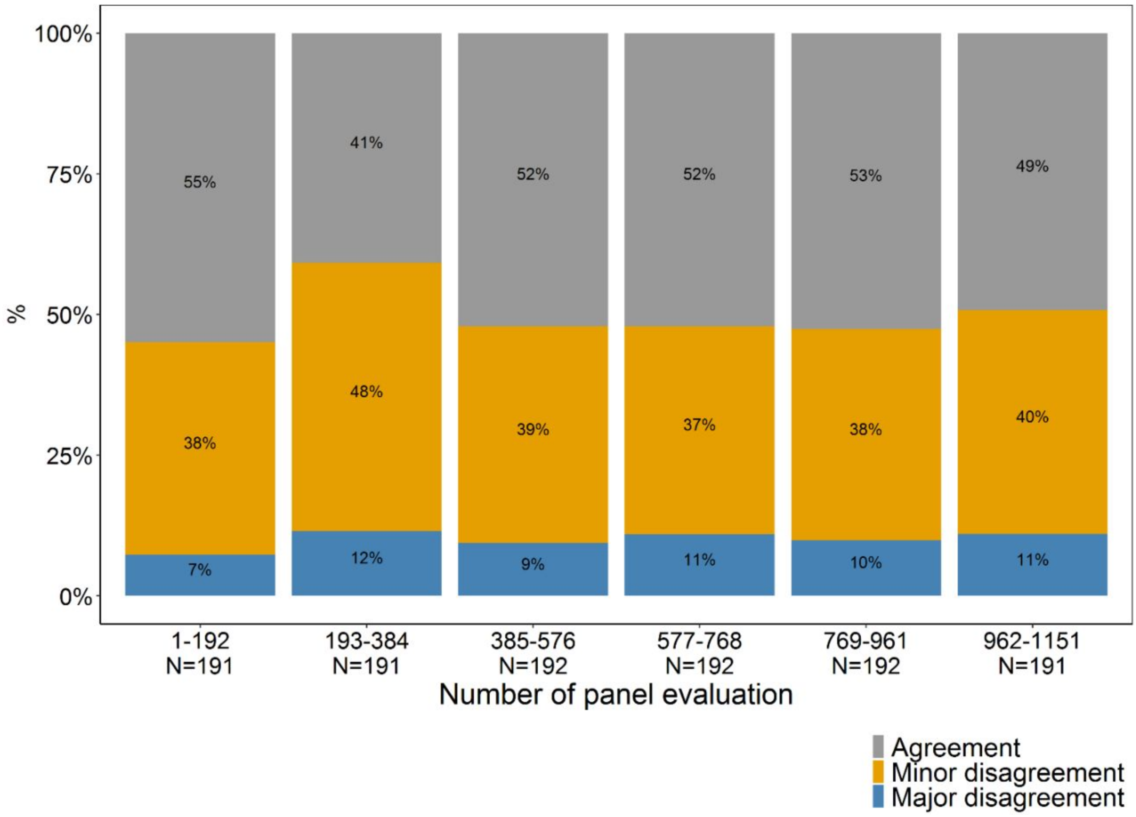

Table S1: Type of local treatment in patients who received complete local treatment.

	N=235
(Extended) hemihepatectomy	23 (9.8%)
(Extended) hemihepatectomy + (combination of) local resection/ablation	30 (12.8%)
Two stage: 1. ablation 2. (extended) hemihepatectomy	2 (0.9%)
Two stage: 1. wedge resection 2. (extended) hemihepatectomy	21 (8.9%)
Two stage: 1. segmental resection 2. (extended) hemihepatectomy	2 (0.9%)
Two stage: 1. combination of local resection/ablation 2. (extended) hemihepatectomy	9 (3.8%)
Two stage: 1. (combination of) local resection/ablation 2. (extended) hemihepatectomy + (combination of) local resection/ablation	9 (3.8%)
Ablation	9 (3.8%)
Segmental resection	9 (3.8%)
Wedge resection	17 (7.2%)
Combination of local resection/ablation	96 (40.9%)
Two stage: 1. (combination of) local resection/ablation 2. (combination of) local resection/ablation	6 (2.6%)
Three stage procedure	2 (0.9%)

Local resection = wedge resection and/or segmental resection.
